# Understanding and Quantifying Molecular Flexibility: Torsion Angular Bin Strings

**DOI:** 10.1021/acs.jcim.4c01513

**Published:** 2024-10-10

**Authors:** Jessica Braun, Paul Katzberger, Gregory A. Landrum, Sereina Riniker

**Affiliations:** Department of Chemistry and Applied Biosciences, ETH Zurich Vladimir-Prelog-Weg 2, Zurich 8093, Switzerland

## Abstract

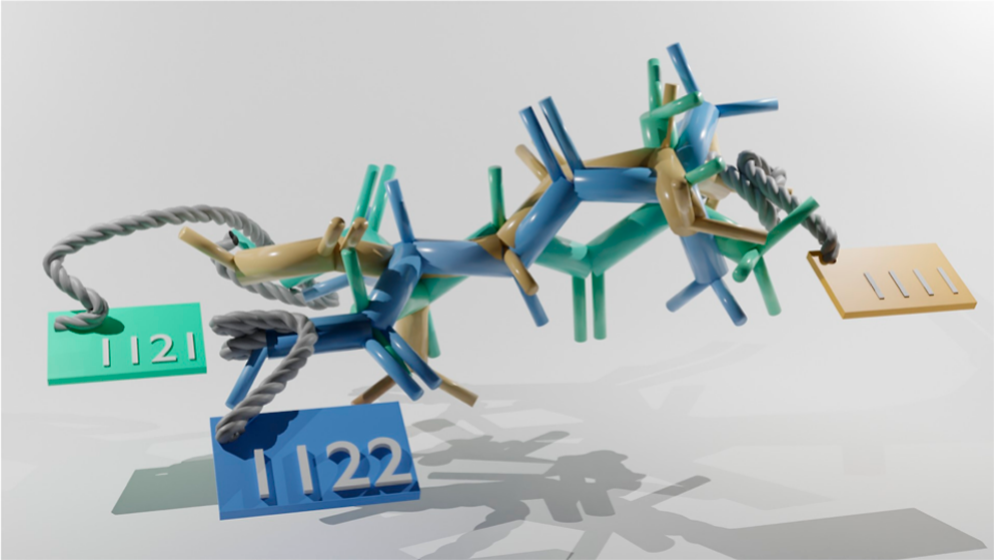

Molecular flexibility is a commonly used, but not easily quantified term. It is at the core of understanding composition and size of a conformational ensemble and contributes to many molecular properties. For many computational workflows, it is necessary to reduce a conformational ensemble to meaningful representatives, however defining them and guaranteeing the ensemble’s completeness is difficult. We introduce the concepts of torsion angular bin strings (TABS) as a discrete vector representation of a conformer’s dihedral angles and the number of possible TABS (nTABS) as an estimation for the ensemble size of a molecule, respectively. Here, we show that nTABS corresponds to an upper limit for the size of the conformational space of small molecules and compare the classification of conformer ensembles by TABS with classifications by RMSD. Overcoming known drawbacks like the molecular size dependency and threshold picking of the RMSD measure, TABS is shown to meaningfully discretize the conformational space and hence allows e.g. for fast checks of the coverage of the conformational space. The current proof-of-concept implementation is based on the ETKDGv3 conformer generator as implemented in the RDKit and known torsion preferences extracted from small-molecule crystallographic data.

## Introduction

Many molecular properties of interest (e.g., the likelihood of a molecule to crystallize^1^ or flexibility–activity relationship information derived from NMR data^2^) are determined by the three-dimensional (3D) structure of molecules. However, at temperatures above absolute zero, most molecular structures are not well described by a single conformation, which makes it necessary to consider a multitude of conformational states. Though intuitive and commonly used, flexibility itself is not easily definable or quantifiable,^3^ and different notions have been introduced focusing on the kinetic, thermodynamic, or structural meaning of the term.^3^ In this study, flexibility will be interpreted as the range of motion of all torsion angles of a molecule and thereby the ensemble of all possible torsion states. In other words, we define flexibility as the factor that determines the size and complexity of the conformational space of a molecule (given solvent, temperature, and pressure).

Understanding the behavior of molecules in a given environment is necessary as some of their properties depend on the conformational states they adopt (e.g., lipophilicity,^4^ passive membrane permeability,^5^ or dipole moment^4^). Although the conformational space is continuous, for the purposes of any kind of analysis it is often discretized by identifying a number of representative conformers, each of which is a substitute for a (potentially large) number of nearby conformers. This discretization requires the selection of a distance or similarity metric between conformers, which may be based solely on the geometry of a conformer (e.g., heavy-atom root-mean-square deviation (RMSD)), a torsion based measure (e.g., torsion-fingerprint deviation (TFD)^6^), or further informed by additional factors like energy (e.g., as used in free-energy^7,8^ or energy-based clustering^9^). With this discretization of the conformational space, the minimum number of conformers needed to describe the ensemble can in principle be deduced. Following this logic, the size of the conformational space of a molecule, and thus its molecular flexibility, can be estimated by generating a large ensemble of conformers and then pruning them based on a chosen distance metric.

One of the most frequently used descriptors to quantify molecular flexibility is the number of rotatable bonds. Though common, this descriptor suffers from a number of drawbacks. Perhaps the largest of these is that it requires a clear, and ideally easily computed, definition of which bonds are rotatable. There are many of such definitions, e.g., the one proposed by Bath et al.,^10^ but not one used by all. Furthermore, the number of rotatable bonds, being constrained to integer values, provides a very coarse-grained view of the size of the conformational space and ignores the fact that different types of bonds have different degrees of rotational freedom. To overcome these challenges, Kier developed the ϕ index,^3^ which provides a continuous description of the flexibility space derived solely based upon information from the molecular graph. While being an improvement in comparison to the number of rotatable bonds, the Kier ϕ does not resolve all issues as it is unable to distinguish stereo- and regioisomers,^11^ and, as we will discuss below, is not particularly effective when used to estimate the size of the conformational space for a molecule.

In this work, we introduce torsion angular bin strings (TABS) to capture the conformational space of a molecule in terms of its torsion angles. We also introduce a new 2D flexibility descriptor, nTABS, that gives the number of distinct TABS for a molecule and thus provides an estimate for the number of representative conformers. Though nTABS is a 2D descriptor (i.e., calculated solely from the molecular topology), it relies upon reference data or parameters generated from 3D information on a large number of molecules (see below). This is similar to other common 2D descriptors like the topological polar surface area (TPSA)^12^ and van der Waals surface area (VSA).^13^ In contrast to Kier ϕ, nTABS is able to account for specified/unspecified stereochemistry and the differences in conformational flexibility in regioisomers. A TABS itself is a vector representation for a conformer reduced to a description of its dihedral angles: Each vector element corresponds to the binned value of the torsion about one rotatable bond in the molecule. The TABS representation discretizes the torsion space and is a form of dimensionality reduction that simplifies the analysis and understanding of conformational ensembles. After this definition, it is also clear that TABS and a torsion fingerprint (TF)^6^ are inherently different as the TFs operate on a continuous space, as well as treating ring contributions as average sums. TFD^6^ itself and TABS are only comparable if a distance metric between two TABS was defined, which has not been done as part of this initial method development.

## Theory

### Common Flexibility Metrics

Before describing our new flexibility metric nTABS, we provide a short overview over two of the most commonly used flexibility metrics: number of rotatable bonds and Kier ϕ index.^3^

### Rotatable-Bond Count

The most common definition of a rotatable bond is a single bond that is not part of a ring connecting two atoms, which each have at least one other nonterminal substituent.^14^ Refinements typically include aspects like ignoring bonds where one atom has only symmetry-equivalent substituents or including bonds in macrocycles. Here, we use the default rotatable bond definition in the RDKit,^15^ described in detail in the Supporting Information S1.

### Kier ϕ Index

As with the rotatable-bond count, Kier treats flexibility as a structural attribute that can be derived directly from the molecular graph.^3^ Kier’s reference point for a perfectly flexible molecule is the infinite chain of carbon atoms with sp^3^ hybridization (Csp^3^), which marks the point where the flexibility index ϕ is defined to be infinite. The definition of ϕ moves on to quantify the extent to which structural features (like having a finite number of atoms, branching, cycles, and the presence of heteroatoms) decrease this perfect flexibility.^3^ Kier defines ϕ using two of the κ shape indices he had previously introduced, ^1^κ^16^ and ^2^κ.^17^^2^κ accounts for the number of atoms and relative cyclicity by counting all 2-bond fragments in a molecular graph,^17^ whereas ^1^κ is the 1-bond fragment count and hence encodes the branching.^16^ An additional factor, α, is introduced to account for the contributions of atom types other than Csp^3^ to the shape.^3^ The normalized shape indices ^1^κ_α_ and ^2^κ_α_ are combined and scaled by the number of atoms *A*, yielding the Kier ϕ index to describe the overall molecular flexibility
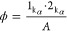
1

### Torsion Angular Bin Strings

**Figure 1:**
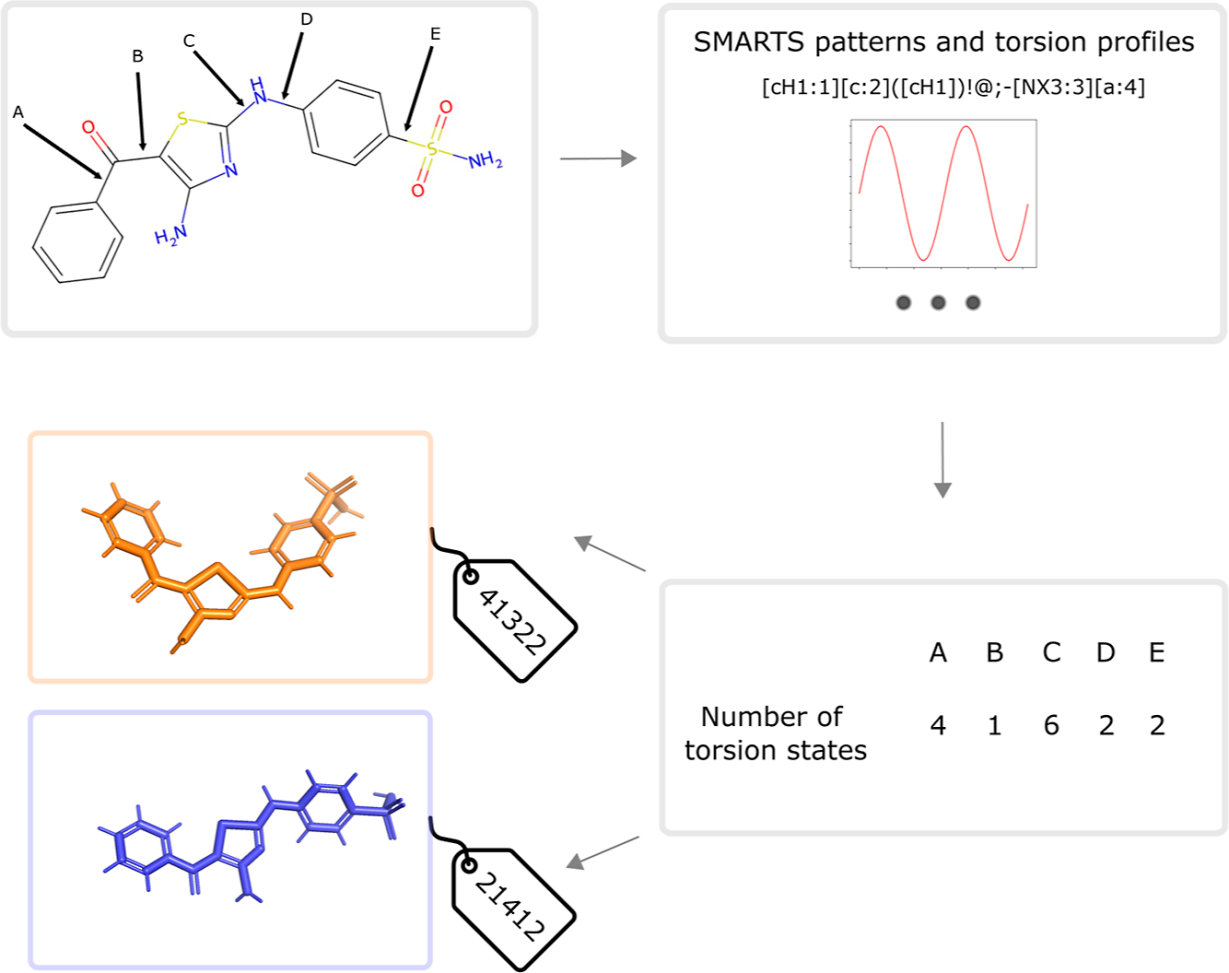
Example of TABS assignment for two example conformers of a molecule.

The TABS for a conformer is a vector with binned values of each of its torsional degrees of freedom. In order to generate a TABS, we developed an approach to determine the number of rotameric states each rotatable bond can adopt and to bin the actual torsion values. Per conformer, this will result in one label with a total length equal to the number of rotatable bonds. As illustrated in Figure 1, the label is obtained from the vector form of the torsion's state numbers.

#### Regular Torsions

In a molecule, each rotatable bond, as identified by a chosen definition, can be associated with a distinct torsion profile. These torsion profiles are influenced by the molecule’s overall structure, giving each dihedral an individual profile. Though each torsion is, in principle, unique, it is possible to assign them to a comparatively small number of archetypes, as for instance introduced by Schärfer et al.^18^ and Guba et al.^19^ The ETKDG conformer generation algorithm^20^ builds upon this work, using a hierarchy of torsion angles that are matched via SMARTS patterns with small-molecule crystallographic data from the Cambridge Structural Database (CSD).^21,22^ Each torsion angle distribution obtained from the CSD was fitted by describing the torsion energies with a Fourier series as commonly used in classical force fields^23^
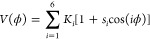
2where *s*_*i*_ ∈ {−1, 1} is the phase shift, and *K*_*i*_ the force constant. Here, the maximum value of *i* is 6, hence the highest multiplicity possibly occurring in the resulting fits is 6.

For TABS, the derivative of each fitted torsion potential from ETKDGv3 was used to determine its multiplicity and define the bins characterizing its possible states. An example is shown in the Supporting Information S3.

Figure 1 shows an example of calculating TABS for a molecule’s conformers: After matching the rotatable bonds to the corresponding experimental torsion profiles using SMARTS, two pieces of information are available: (i) how many torsion states are in theory possible for each identified dihedral, and (ii) the torsion bin values associated with those states. Generating the graph automorphisms for the molecule (substructure matches of the molecule onto itself) allows us to determine which TABS are symmetry equivalent at the topological level. As the example molecule has no automorphisms, we can calculate nTABS by taking the product of the number of possible states for the torsions A to E. Thus, nTABS is 96 for this molecule.

### Highly Correlated Torsions in Substructures

Torsions in real molecular systems can be correlated, but TABS make the assumption that dihedral angles are independent of each other to allow for the counting of states based on the aggregated torsion profiles. While a small overestimation of the number of possible conformers in chains is accepted, this is not defensible when considering ring structures. A quick estimation of the overcount shows the importance of taking correlation into account for rings: Given the SMARTS pattern matching the dihedrals in an aliphatic six-membered ring, there would be three bins per bond, which leads to 3^6^ = 729 possible combinations of the six bits of the TABS for the ring. However, a categorization into boat, chair, and twist boat (with the respective transition states, plus the extreme case of a planar ring) is much more accurate, highlighting why a reduction for small and medium-sized rings as well as macrocycles was needed.

### Small and Medium-Sized Ring Systems

Small and medium-sized rings (ring size = 3–11) are still described in TABS by the states of their individual torsions. However, when calculating nTABS, we take the high correlation between ring dihedrals into account by using a single number: the maximum number of states for the aliphatic ring of that size known from literature.

The most flexible, but also most symmetrical, case for the rings was selected as the point of reference, which is the corresponding cycloalkane. For each of their conformations as reported in literature, the torsion angle ensembles were obtained and analyzed for symmetries to obtain information about how many different cases of one conformation could occur should asymmetries be present. As an example, the chair conformation of cyclohexane exhibits an torsional angle pattern of two different repeating values, which can be expressed in numbers as 121212. If no substituents or heteroatoms, which could potentially break symmetries, are present, both chair conformations are indistinguishable (121212 = 212121). When hetero atoms and/or substituents break symmetry, 121212 and 212121 become distinguishable and hence two chairs can be identified, resulting in two different chair conformations connected by the chair flip. This analysis was performed for ring sizes ranging from three to 11 (Table 1).

**Table 1:** Number of Conformational States Considered for nTABS Based on Ring Size as Derived from Literature References

ring size	maximum number of states	literature reference
3	1	
4	3	(24)
5	11	(25)
6	15	(26)
7	29	(27)
8	45	(27)
9	115	(27)
10	181	(27)
11	331	(28)

Additionally, it should be noted that in the TABS procedure, no ordering by neighboring bonds is enforced, meaning that the symbolically written 121212 for the two present unique angle values in the chair conformation of cyclohexane could present in a permuted form when running the TABS code.

### Macrocycles

The ring strain of aliphatic rings decreases with their size, hence from a ring size of 12 onward, their torsion profiles resemble those of their linear counterparts. Therefore, when generating TABS, macrocycles will again be classified by the states of their dihedrals, in line with non-ring torsions. As this still leads to a substantial overestimation of the accessible conformational space, we introduced a correction factor when counting the number of possible TABS. Details of the derivation of the correction factor are given in the Supporting Information S4. The corrected upper bounds used for macrocycles in the nTABS calculations are displayed in Table 2. As the intended usage of TABS in its current form is for small to medium sized drug-like molecules, larger macrocycles (>16) are not recommended to be analyzed with it.

**Table 2:** Maximum Number of States Considered for Macrocycles in nTABS Calculation

ring size	maximum number of states
12	16549
13	44934
14	122002
15	331251
16	899394

#### Influence of Topological Symmetry

**Figure 2:**
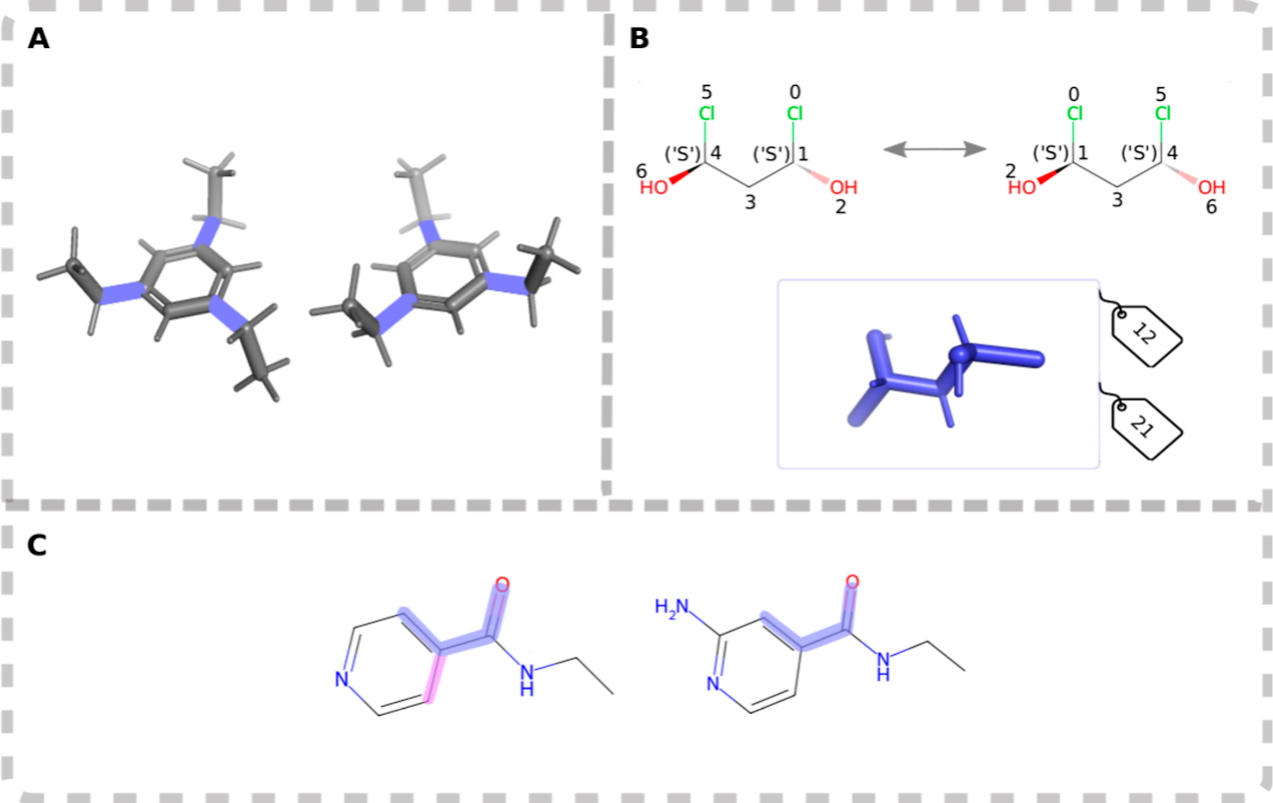
(A) Example of 1,3,5-triethylbenzene to illustrate why local symmetry is not included in TABS and nTABS. (B) Example for the graph automorphism used to detect global topological symmetry and the following TABS equivalence. (C) Example of local symmetry not included in TABS or nTABS, magenta marks the atom rank equivalent selection.

As with most properties derived from molecular structure, topological symmetry^29^ has a non-negligible influence on TABS and nTABS. In this context, symmetry contributes both at the global (full molecule) and local (bond environment) levels. Local symmetry, where at least one of the two atoms constituting a bond is connected to multiple symmetry-equivalent atoms, intuitively should result in a reduction in the number of distinguishable torsion states. In simple cases, where the local symmetry is isolated and no other global symmetries exist, this results in different bins of the torsion profile being equivalent. However, the integration of local symmetry in the algorithms for TABS and nTABS is problematic. Taking 1,3,5-triethylbenzene as an example, there are three torsions identified, marked in blue in Figure 2A, each of which has a multiplicity of two. Each of the three torsions of this molecule includes a topological symmetry group with the atoms in the phenyl ring neighboring the atom that connects the ring to the substituents. This implies a mirror plane, which reduces the torsional space of each bond by a factor of 2. Naively taking two bins to be the same for torsions with a multiplicity of two would result in the bins being merged, yielding a nTABS of 1. This is clearly not the case, as can be seen from the two different conformers depicted in Figure 2A. This simple example shows that the decision on whether a local topological symmetry results in a reduction in the number of bins in a torsion profile cannot be made without considering the overall molecular symmetry. The two overall configurations in Figure 2A can be labeled as [above, above, below] and [above, above, above].

Additionally, the direct inclusion of the local symmetry is a challenge as the extracted torsion profiles and their fits are an abstraction layer, which can result in possible mismatches in the identified multiplicity and symmetry. Not accounting for local symmetry does result in inaccuracies. For example, the molecules in Figure 2C will be assigned the same nTABS even though the symmetry of the six-membered ring in the molecule on the left would lead us to expect it to have half as many conformers. However, as we use nTABS to provide an upper limit on the size of a molecule’s conformational space, we do not see this overestimation as a significant problem.

Global topological symmetry is important when different rotatable bonds are symmetry equivalent. For TABS, this translates to different bits within one TABS describing the same rotatable bond, leading to permutations of TABS that correspond to the same 3D structure. This symmetry is detected by identifying and marking automorphisms on the molecular graph and taking chirality into account. An example of such a global symmetry is shown in Figure 2B, where one possible alternate mapping is identified. With (0,1,3,4) and (1,3,4,5) as the two dihedrals spanning the conformational space, each TABS for this particular molecule consists of two digits. Analyzing the symmetry reveals that a permutation of these two digits corresponds to equivalent structures. In these cases, we choose the arrangement of digits that produces the TABS with the lowest integer value, here 12.

### Calculating nTABS

The number of naive TABS (nTABS_naive_) for a given molecule can be derived in the most intuitive way as the product of all multiplicities
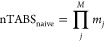
3with *M* being the number of dihedrals contributing to the TABS.

It can also be written as the sum of all possible representative TABS *r*_*i*_ multiplied by the size of the permutation set for each case *p*_*i*_ for *N*_poss_, the count of possible distinct cases.
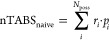
4When symmetry is present, the *p*_*i*_ are larger or equal to 1. As always one representative is selected, the number of possible TABS (nTABS), representing the number of TABS after symmetry reduction, is the sum over *r*_*i*_.
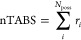
5When no topological symmetry is present, nTABS_naive_ is equivalent to nTABS. In the presence of highly correlated substructures (i.e., small/medium sized rings and/or macrocycles), the multiplicities of the correlated dihedrals are represented by one single contribution for the entire highly correlated substructure. The exact values for these contributions are listed in Tables 1 and 2.

### Notes on Comparing TABS and nTABS

We would like to emphasize that only TABS originating from the same molecule are comparable and that both TABS and nTABS are dependent on the torsion profiles used. Furthermore, it has to be stressed that the torsion profiles in the current TABS implementation are derived from small-molecule crystal structures. A different set of torsion profiles would need to be used in order to generate TABS that are optimally suited for different environments like solution or gas-phase. This will be an area of future research.

We also want to note that the atom numbering of a molecule is not canonicalized as part of the TABS code, i.e., it is possible to arrive at two different TABS for equivalent conformers of the same molecule if they differ in their atom ordering. The easiest way to resolve this is to renumber the atoms in one of the molecules to make the atom numberings equivalent. The RDKit provides code to do this and a usage example is provided in the TABS GitHub repository. In general, for the analysis of conformer ensembles, we recommended to work with one RDKit molecule containing all of the conformers in the ensemble.

## Methods

### Data Set

The chosen data set for the proof of concept was the Platinum data set generated by Friedrich et al.^30^ As shown in ref (30), the curated small-molecule structures from the PDB^31^ are representatives for drug-like molecules. The version used here was the Platinum 2017_01, which includes a total of 4548 compounds. Out of those, 2166 contain aliphatic small to medium-sized rings and 31 contain macrocyclic substructures. 3062 of the 4548 molecules exhibit topological molecular symmetry. For the purposes of the analysis in this study, we used nTABS to further decompose the Platinum set into three subsets: low flexibility molecules (nTABS < 500), medium flexibility molecules (500 < = nTABS < 10,000), and high flexibility molecules (nTABS > = 10,000).

### Note on Calculating TABS with ETKDG

In order to generate a TABS for a molecule, we need to be able to bin the torsion profile of every rotatable bond. In this work, we used the ETKDGv3^20,32^ pattern library (including small-ring torsions), which is based on the SMARTS patters in ref (19). As this library does not cover all bonds assigned to be rotatable using the RDKit’s definition,^15^ we need to calculate multiplicities and bins also for these additional rotatable bonds. The additional dihedrals were assigned a multiplicity of six as a default option, binning the torsion profile arbitrarily at 30, 90, 150, 210, 270 and 330°.

### Analysis

**Figure 3:**
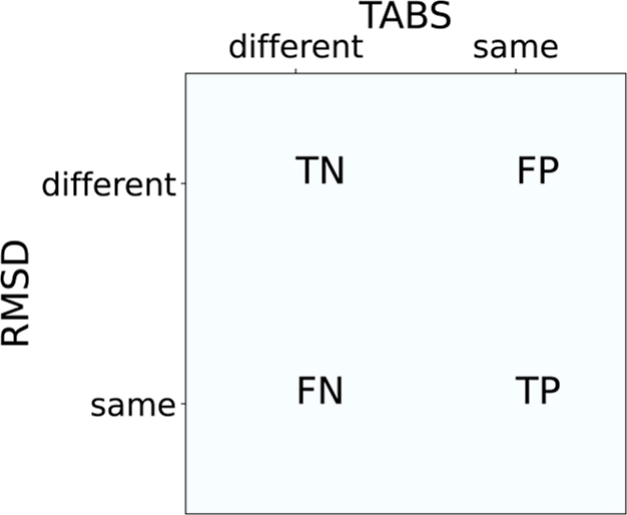
Confusion matrix for the comparison between the TABS and heavy-atom RMSD categorization (TN: true negatives, TP: true positives, FN: false negatives, FP: false positives).

For the comparison of the categorization of conformers with TABS and with heavy-atom RMSD, we calculated confusion matrices. As the categorization for two conformers of an ensemble being the same or different is currently most commonly understood by using a RMSD threshold, the TABS categorization of two conformers into the same or different was compared to it. To account for the dependency of an appropriate RMSD threshold on the size of a molecule, thresholds in the interval [0.2, 2.4] Å were scanned. At each RMSD threshold, a confusion matrix was calculated as shown in Figure 3 and an overall confusion matrix per RMSD threshold obtained by summing up all molecules’ confusion matrices. At each chosen threshold, the RMSD categorization was assumed as the ground truth and the TABS categorization as the prediction. Each confusion matrix was analyzed for two metrics, the positive predictive value (PPV, also known as precision) and the negative predictive value (NPV)
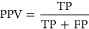
6
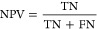
7

## Results and Discussion

The meaningfulness of TABS to discretize and sort the conformational space of molecules is assessed by comparing the grouping by TABS to the commonly used heavy-atom RMSD.

### Comparing TABS of Ring-Containing Conformers

**Figure 4:**
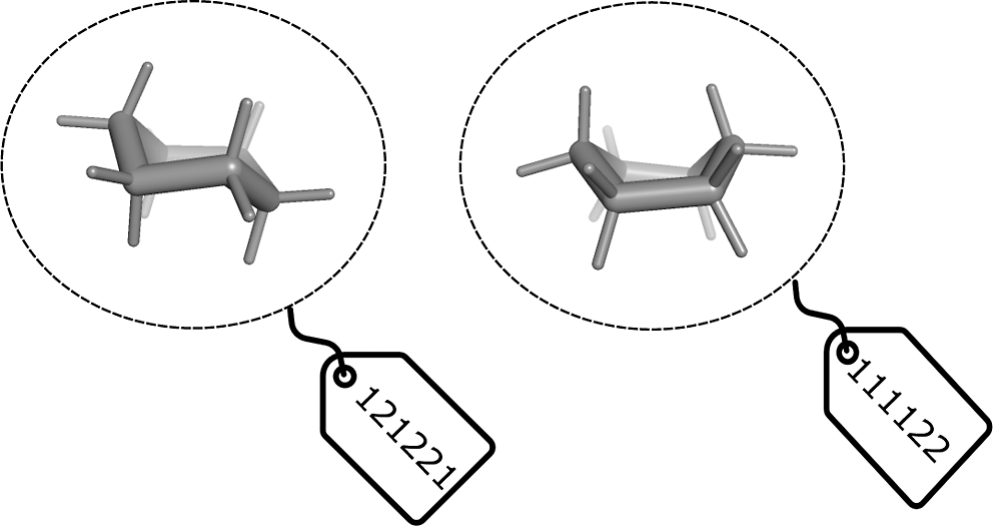
Comparing two conformers of cyclohexane, chair (left) and boat (right), and their assigned TABS.

As an example, two representative conformers of cyclohexane, namely the chair and boat conformations, were compared in their RMSD and TABS labels. Figure 4 shows the two conformations and their assigned TABS, illustrating that the two are marked as different by TABS. In contrast, the RMSD between the two conformers is 0.33 Å, i.e., they would be grouped together by the commonly used RMSD threshold of 0.5 Å.

### Comparing Categorization with TABS versus RMSD

**Figure 5:**
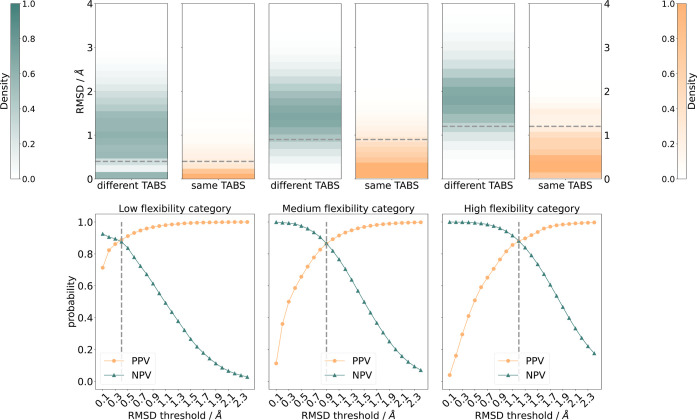
(Top): Distribution of the RMSD values for ensembles of the molecules from the Platinum set^30^ with the same TABS value (orange) and different TABS values (green), split into low (left), medium (middle), and high flexibility (right). The RMSD value at the PPV/NPV intersection point in the bottom row panels is marked as a dashed gray line. RMSDs larger than 4 Å are not displayed as they only occurred for very few examples in the different TABS category. (Bottom): Positive predictive values (PPV, orange) and negative predictive values (NPV, green) as a function of the RMSD threshold sorted into the different flexibility categories according to the introduced nTABS categorization.

Estimating the quality of the TABS categorization is not straightforward as no true reference exists. Here, we chose to use the heavy-atom RMSD to qualitatively validate our approach. As we would expect two conformers with equal TABS to be similar to each other, we would expect a low RMSD value for this pair. Similarly, we would expect conformers with different TABS to have higher RMSD values. The RMSD distributions of different and equal TABS for molecules with low, medium, and high flexibility are shown in Figure 5. As expected, these distributions follow the predicted trend with much lower RMSD values between conformers in the same TABS category. To quantify these results, confusion matrices were constructed using fixed RMSD cutoffs between 0.2 and 2.4 Å to classify two conformers as equal or unequal. The TABS categorization of whether conformers are the same or different remains the same regardless of how the RMSD-based categorization changes.

The results for each RMSD cutoff are shown in Figure 5. The larger the RMSD threshold was set to, the more conformer pairs were considered the same, leading to a decrease in the number of false positives (FP) and true negatives (TN) along with an increase in the number of true positives (TP) and false negatives (FN). This leads directly to the observed overall trend of positive predictive value (PPV, eq 6) steadily increasing and negative predictive value (NPV, eq 7) steadily decreasing in all three categories. To achieve the best agreement between TABS and RMSD, both the PPV and NPV values should be maximized. This leads us to choose the RMSD threshold for each category at the point where the curves for PPV and NPV cross. The intersection point moves toward larger RMSD threshold values from low to high flexibility, a finding that is in line with the well-known size dependence of RMSD thresholds.^6^

The same analysis was performed with the TFD method using the threshold of 0.2 proposed by Schulz-Gasch et al.^6^ The much lower NPV and PPV values in Figure S7 in the Supporting Information show that the two metrics RMSD and TFD disagree much more for the molecule ensembles in the Platinum set than TABS and RMSD. While the classifications based on TABS and RMSD were reaching NPV and PPV values of over 80% for all three flexibility categories, the values in the comparison RMSD versus TFD do not exceed 60%.

### Correlation of Pruned Ensembles with Descriptors

**Figure 6:**
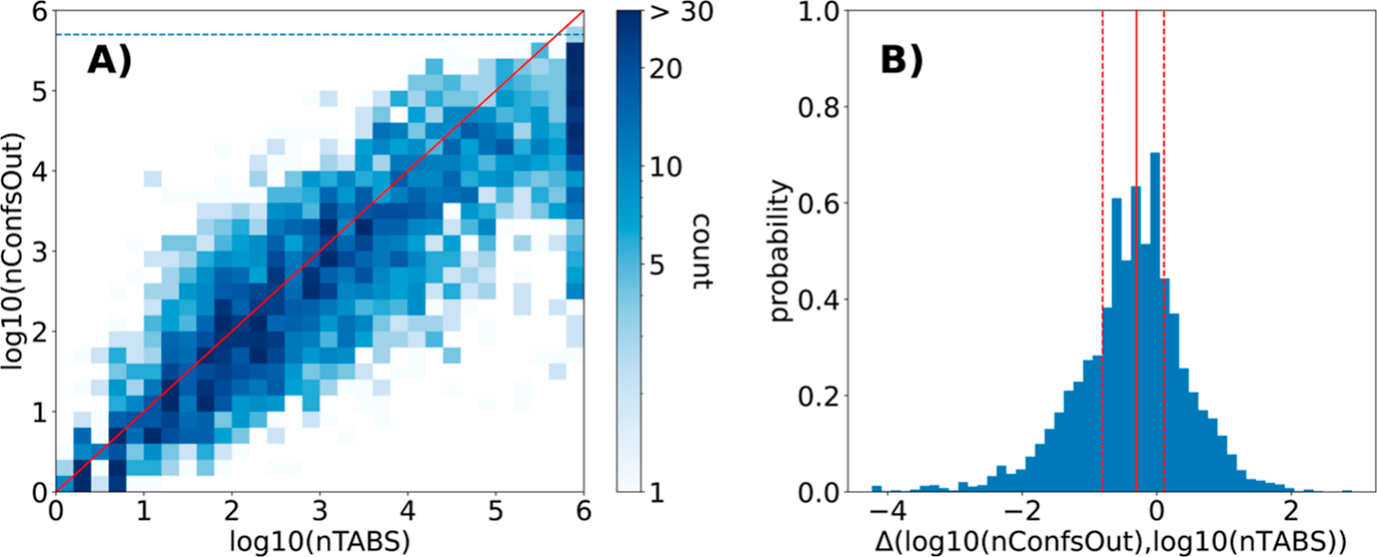
(A) Correlation between the number of conformers produced by ETKDGv3 (including small-ring torsions) with pruning of 0.5 Å requesting 500′000 conformers and nTABS (both on a logarithmic scale). (B) Histogram of the difference between the two metrics in (A). Marked in red are the 25% percentile, the median and the 75% percentile.

In contrast to other flexibility metrics (e.g., rotatable bonds or Kier ϕ index), nTABS allows for a direct estimation of the upper bound of the number of possible conformers, which is a desirable feature in the field of conformer generation. To validate the accuracy of nTABS as an estimate for molecular flexibility, large conformational ensembles (*N*_input_ = 500′000) were generated using RMSD pruning (*R*_cutoff_ = 0.5 Å). The size of the pruned ensemble comes closest to a true measure of flexibility (but is computationally relatively expensive compared to simple metrics like the number of rotatable bonds or Kier ϕ index). Figure 6A shows the comparison of the size of the pruned ensemble with nTABS (both on log-scale). The general correlation demonstrates a good agreement between the two metrics and the slightly negative median deviation of −0.3 log units between the estimated and observed ensemble sizes supports the notion of nTABS providing an upper estimate on the ensemble size (Figure 6B).

With nTABS primarily intended as an upper limit for the number of conformers, molecules with ensemble sizes significantly above the nTABS prediction were analyzed in greater detail. These deviations were mainly due to limitations of the underlying torsion profiles. A detailed summary of the identified issues is provided in the Supporting Information S5.

### Performance Measurements

The calculation time of the TABS algorithm scales linearly with the number of conformers. This is significantly better than RMSD-based clustering, which scales with the number of conformers squared. As an illustration, the timings for the calculation of the TABS labels for the conformational ensemble of cyclohexane with the tabs. GetTABSMultipleConfs function takes 1–1.5 ms per conformer on a standard workstation. The exact timings are given in the Supporting Information S8.

Note that in the current implementation, TABS and nTABS calculation is also dependent on the complexity of the symmetry present in the molecule.

## Conclusions

With TABS, we have introduced a compact and efficient new representation for molecular conformers, which reduces them to their torsion space, allowing for quick analysis and grouping of the torsion space covered by a given conformer ensemble. Furthermore, the nTABS descriptor provides a straightforward upper estimate of the size of the conformational space of a molecule.

When applying nTABS to the Platinum set, TABS generally showed very good agreement with heavy-atom RMSD while still allowing important structural changes like small-ring conformations to be distinguished. The related nTABS, which counts the number of possible TABS that can be formulated for a molecule, provides a direct estimation for the upper bound of the size of the conformational space. Comparing nTABS with the size of a large RMSD-pruned ensemble of the molecule (number of conformers = 500′000 and RMSD threshold 0.5 Å), a good correlation was observed.

We provide easy-to-use Python code that allows TABS and nTABS to be used in any cheminformatics project. Future work will include improved sets of torsional profiles and a more detailed consideration of subgraph isomorphism.

## Data Availability

The code used to perform this study is open source and available on GitHub (https://github.com/rinikerlab/TorsionAngularBinStrings).

## Supporting Information Available

Sections of the Supporting Information (S1) Rotatable-Bond Count in the RDKit, (S2) RDKit ETKDGv3 Conformer Generator, (S3) Torsion Profiles and Binning, (S4) Aliphatic Rings in TABS, (S5) Correlation between nTABS and Heavy-Atom RMSD, (S6) Correlation between nTABS and Kier ϕ, (S7) Classification Comparison between Heavy-Atom RMSD and TFD, (S8) Performance Measurements (PDF)
