# Supplementary material for: Understanding and Quantifying Molecular Flexibility: Torsion Angular Bin Strings
Source: J Chem Inf Model. 2024 Oct 10;64(20):7917–24. doi: 10.1021/acs.jcim.4c01513 (PMC11523068; doi:10.1021/acs.jcim.4c01513)
Supplement: Supplementary file 1 — ci4c01513_si_001.pdf [file ci4c01513_si_001.pdf]

SUPPORTING INFORMATION
Understanding and Quantifying Molecular Flexibility: Torsion Angular Bin Strings

Jessica Braun,[a] Paul Katzberger,[a], Gregory A. Landrum,[a]
and Sereina Riniker*[a]

[a] *Department of Chemistry and Applied Biosciences, ETH Zürich, Vladimir-Prelog-Weg 2, 8093 Zürich, Switzerland. E-mail: sriniker@ethz.ch*

Contents

S1 Rotatable-Bond Count in the RDKit **S2**

S2 RDKit ETKDGv3 Conformer Generator **S2**

S3 Torsion Profiles and Binning **S3**

S4 Aliphatic Rings in TABS **S3**
S4.1 Small and medium sized rings S3
S4.2 Macrocycles S5

S5 Correlation between nTABS and Heavy-Atom RMSD **S7**

S6 Correlation between nTABS and Kier ϕ **S8**

S7 Classification Comparison between Heavy-Atom RMSD and TFD **S9**

S8 Performance Measurements **S10**

S1 Rotatable-Bond Count in the RDKit

In the RDKit code, rotatable bonds are identified by matching the following SMARTS pattern [1]

```
"[!$(*#*)&!D1&!$(C(F)(F)F)&!$(C(Cl)(Cl)Cl)&!$(C(Br)(Br)Br)&!$(C([CH3])("
"[CH3])[CH3])&!$([CD3](=[N,O,S])-!@[#7,O,S!D1])&!$([#7,O,S!D1]-!@[CD3]="
"[N,O,S])&!$([CD3](=[N+])-!@[#7!D1])&!$([#7!D1]-!@[CD3]=[N+])]-,:;!@[!$"
"(*#*)&!D1&!$(C(F)(F)F)&!$(C(Cl)(Cl)Cl)&!$(C(Br)(Br)Br)&!$(C([CH3])(["
"CH3])[CH3])]"
```

The pattern can be translated to rules:

1. The bond must be an acyclic single or aromatic bond.
2. Neither atom involved in the bond can be involved in a triple bond.
3. Neither atom involved in the bond can have only one explicit connection.
4. Neither atom involved in the bond can be the central atom of a CF_3, CCl_3, CBr_3, or t-Bu group.
5. The atom on the left side cannot be part of a C(=X)(X′) pattern with X=N,O,S and X′=N,O,S.
6. The atom on the left side cannot be the first N in a NC=N^+ group.

This implementation currently does not account for the case that a chain of single-triple-single bonds or repetitions of this pattern are rotatable.

S2 RDKit ETKDGv3 Conformer Generator

As the notion of flexibility here is directly related to the torsion states of each dihedral, the underlying torsion profile of each dihedral needs to be identified when presented with a new molecule. For small molecules, their preferred torsional states in crystalline form can be derived from crystal structure databases, e.g., the Cambridge Structural Database (CSD)[2],[3], which was analyzed using the hierarchical SMARTS torsion patterns by Schärfer *et al.* [4] and Guba *et al.* [5] The hierarchical ordering of the SMARTS patterns, which encode the chemical environments in different levels of detail, defines the rules that are applied [4]. The ETKDGv3sr conformer generator is based on these experimental torsional-angle preferences, which are informed by potentials fitted to the crystal torsional-angle distributions associated with the previously described SMARTS patterns [6, 7]. It also expands the SMARTS pattern list with an additional one for flat aromatic rings (i.e., forcing the torsion value to either 0 or 180) [6]. The most recent curated list can be found in the official RDKit repository [8].

In order to have the most accurate results, the torsion profiles used to define the bins for TABS should be representative of the actual conformational space spanned by the ensemble that is analyzed. Therefore, we use here the same torsion profiles used for the experimental torsion (ET) terms in the RDKit's ETKDGv3sr conformer generator [6, 7]. Together with the knowledge (K) terms [6], they are used to in the final minimization step of the distance geometry (DG) algorithm. Note that due to the weighing of the different contributions in this final minimization step, a torsion profile for a specific molecule can diverge from the reference torsion distributions, e.g., steric clashes could prevent the population of a certain bin.

S3 Torsion Profiles and Binning

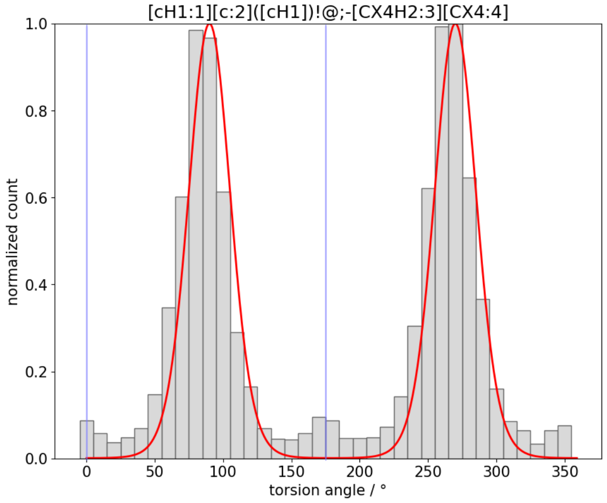

Figure S1: Torsion profile for CSD query results for one SMARTS pattern; red line: fitted functionals, vertical lines: indicate binning

S4 Aliphatic Rings in TABS

S4.1 Small and medium sized rings

The ring classification scheme as discussed in the Method section was implemented and tested by comparing the intra vs inter TABS RMSD.

Figure S2 shows the TABS obtained for an ETKDGv3 ensemble of 100 conformers, not switching on the small ring torsions since ETKDGv3sr does not produce any boats. Sticking to the established names of the conformations of cyclohexane [9], the TABS shown in Fig. S2 are identified as 121221 being chair conformations, 111222 halfboats, 111122 boats, and the remaining ones transition states (variations of twistboat, halfchair).

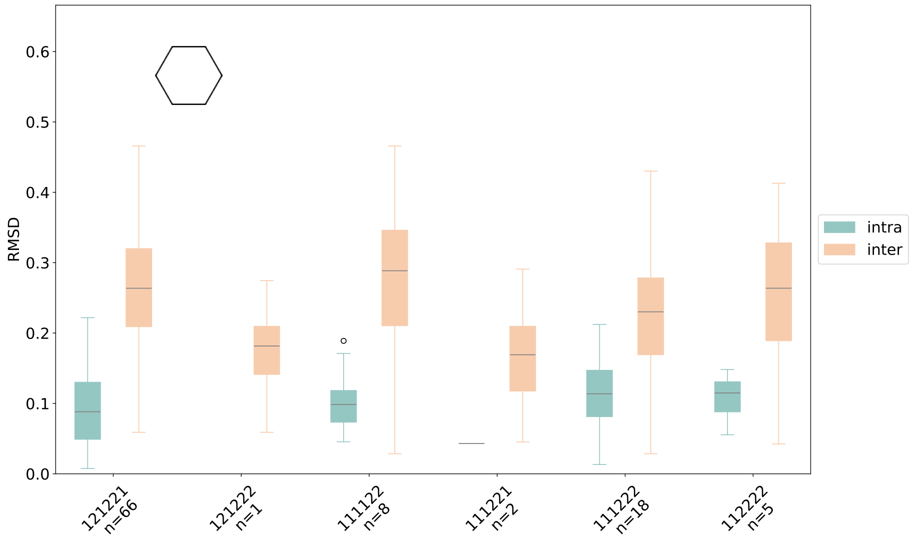

Figure S2: Intra and inter RMSD for an ETKDGv3 ensemble of 100 conformers of cyclohexane with switched off small ring torsion preferences

For all different TABS assigned to conformers of the ensemble, the intra RMSD is smaller than the inter RMSD, which indicates that the labelling does agree with a grouping of the conformers by RMSD. Fig. S2 however also shows that the RMSDs for a medium sized ring like cyclohexane are small in general (conformations with a RMSD of 0.5 Å or less are commonly considered to the be same).

For larger molecules with substituents, the difference in RMSD is easier to detect, therefore the example of ethylcyclohexane was also analyzed in Fig. S3.

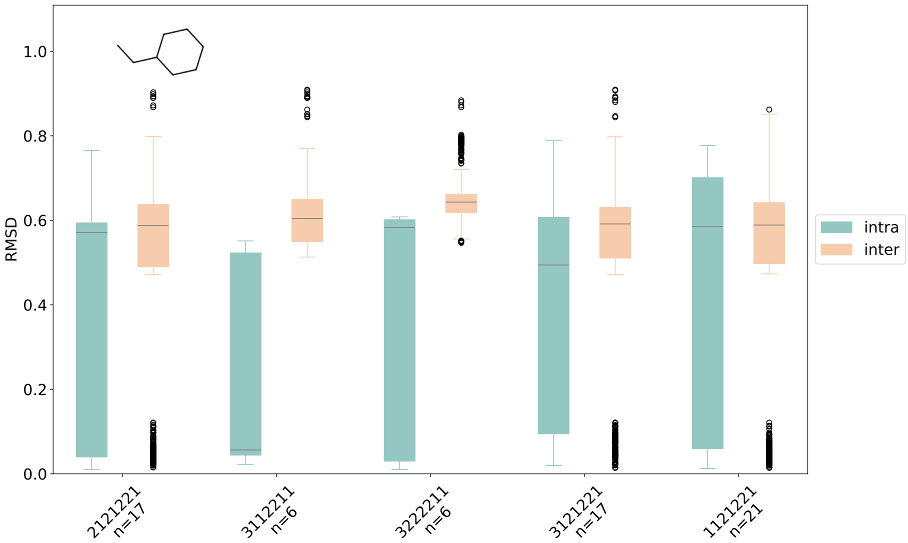

Figure S3: Intra and inter RMSD for an ETKDGv3 ensemble of 100 conformers of ethylcyclohexane with switched on small ring torsions, for readability only TABS populated with more than 5 conformers are shown

S4.2 Macrocycles

For the correction factor, a study on ensembles ($N = 1000000$) of cycloalkanes of sizes 5 to 15 was conducted. For each found conformation, the corresponding TABS was assigned and also in this special case the digits were ordered by their occurrence in the molecular graph. In a second step, as it was known from the graph isomorphism and the enforced ordering that a renumbering of the atoms would correspond to a circular permutation of the TABS, each TABS was complemented by the list of found circular permutations that would correspond to the exact same state. With that, the most symmetric case is used to emulate the most asymmetric case as all the TABS permutations are written out explicitly. This was done to ensure that the upper bound for nTABS was estimated. The results of the study are reported in Fig. S4.

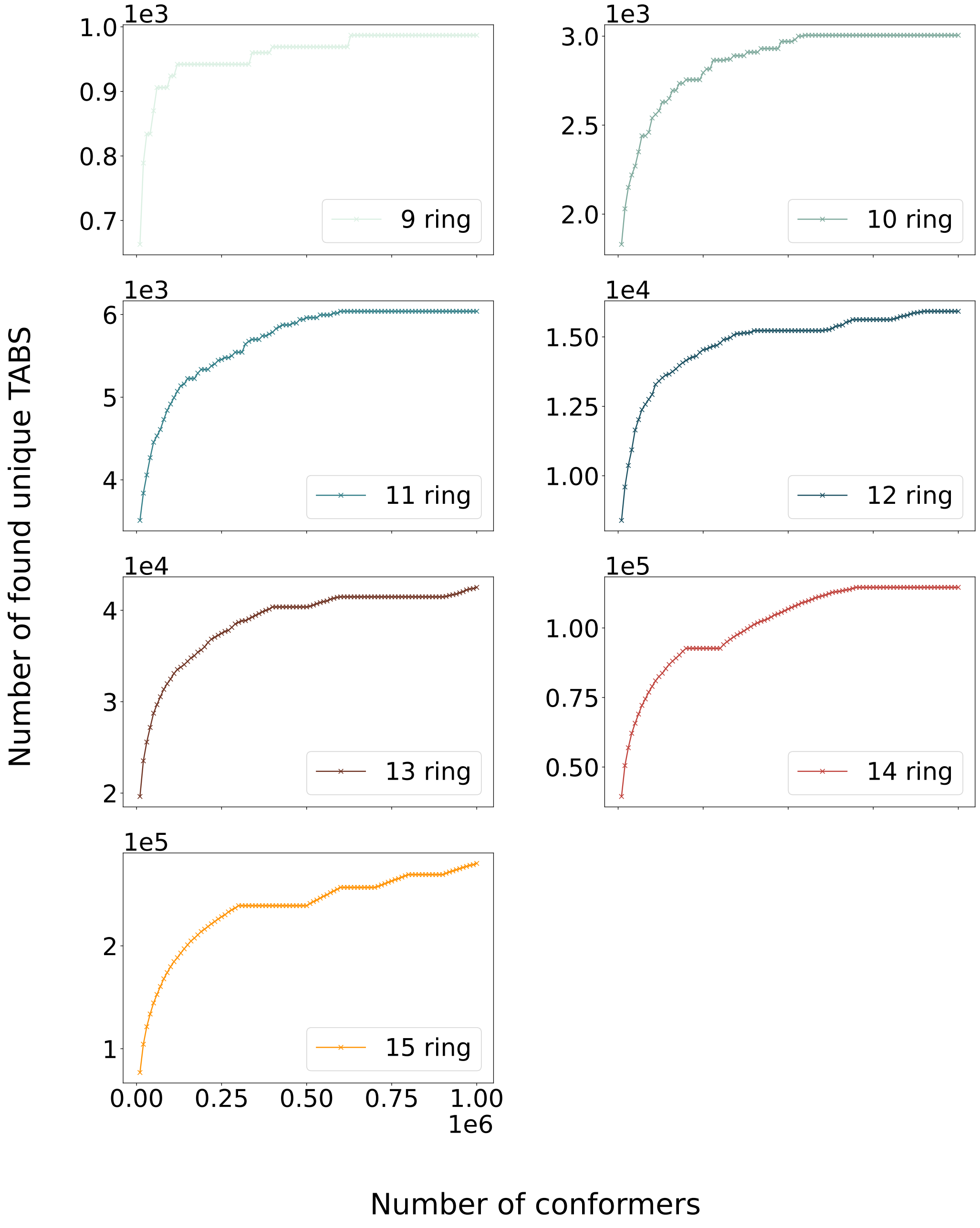

Figure S4: Convergence study for the number of conformers expected when pruning the ensemble by using TABS

Using the estimated converged values shown in Fig. S4, an empirical fit was applied as displayed in Fig. S5.

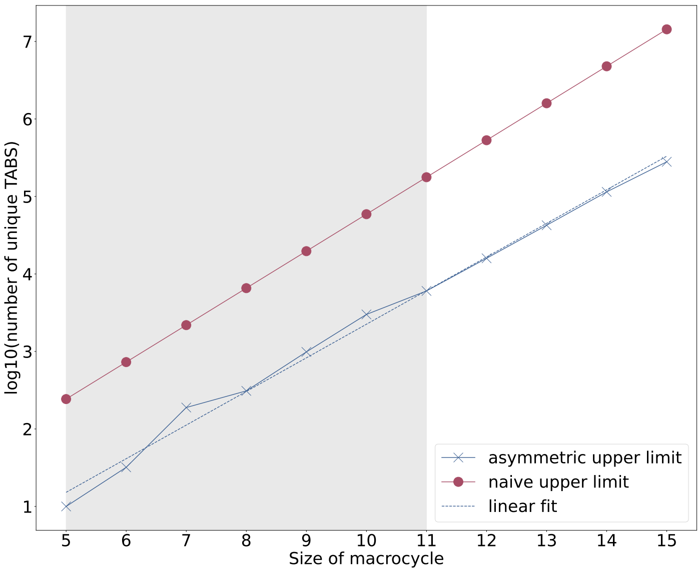

Figure S5: Correction factor analysis for upper limit of expected total number of TABS, explicit consideration of cyclic permutations, grey shading: aliphatic ring sizes that are not considered a macrocycle, but that are shown for the purpose of the fit ($y = 0.43379199 \cdot x - 0.98672204$)

S5 Correlation between nTABS and Heavy-Atom RMSD

As nTABS was introduced as the upper limit for the number of conformers making up the ensemble, large deviations above the nTABS prediction had to be analyzed in more detail. Multiple reasons could be identified that caused the deviations. One being that the pattern of single bond, triple bond, single bond or multiples of it are not recognized as a dihedral torsion and therefore not assigned a torsion profile or a TABS bit. The integration of a general detection of this pattern as part of the rotatable bond count function in RDKit is planned. As TABS and nTABS is based on all rotatable bonds identified by ETKDG and the rotatable bond count, it will resolve this particular issue. Checking additional outliers, the quality of the experimental torsion fits also turned out to be problematic as they are dealing with the differentiation between noise and additional peaks on a case by case basis, sometimes under-counting the number of present peaks. Another factor observed as a cause for outliers is the last minimization step in the ETKDGv3 algorithm: All contributions from the knowledge terms and the experimental torsions alongside all other constraints are optimized within a set maximum number of 300 steps. Initial experiments for observed outliers showed that an increase of the number of maximum steps to 3000 resulted in a decrease of the number of conformers in the pruned ensemble indicating that the minimization did not converge within the 300 steps.

S6 Correlation between nTABS and Kier ϕ

When analyzing results according to their flexibility, it is common practice to categorize according to low, medium and high flexibility by a flexibility descriptor. For the number of rotatable bonds low flexibility is assumed for less or equal to 5, medium for 5 to 9 and high flexibility for equal or more than 10. For nTABS, all molecules with a nTABS of less or equal to 500 are considered of low flexibility, all within 500 to 10000 as medium flexible and all above 10000 as highly flexible. The Platinum dataset was analyzed for the number of molecules present in the different flexibility categories according to nTABS. The relation between the defined categories and the heavy atom count is shown in Fig. S6.

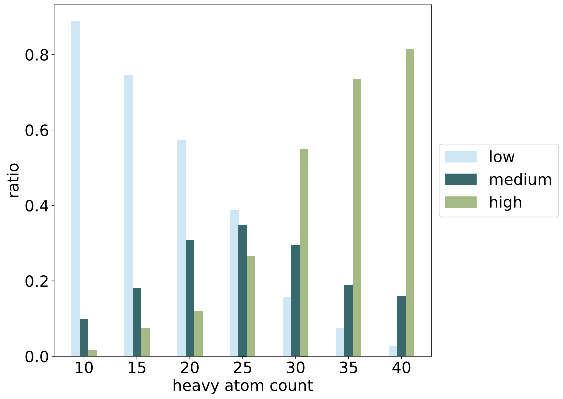

Figure S6: Number of molecules from the Platinum dataset in the different flexibility categories according to the flexibility descriptor nTABS and the ratio of the distribution within the flexibility category to the overall heavy atom count distribution, low flexibility category: 2161 molecules, medium flexibility category: 1141 molecules, high flexibility category: 1246 molecules

Showing in Figure S7, the Platinum set was analyzed with all three previously described descriptors indicating flexibility. For the nTABS metric, the possible value range is $[0, 1000000]$ with the maximal value of 1000000 indicating that the conformer space is equal or greater than 1'000'000. Hence, for the purpose of comparability of the plots, the 360 cases for which nTABS had a value of 1000000 were excluded from the number of occurrences vs. np.log10(nTABS) plot in Figure S7.

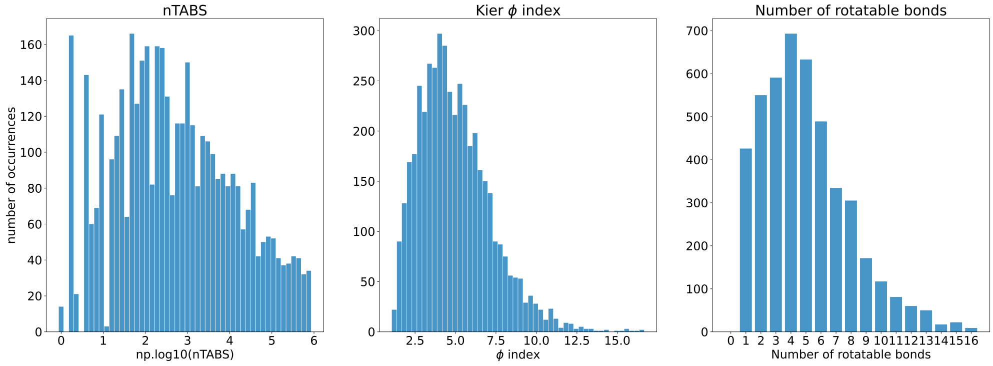

Figure S7: Comparison between the three metrics on the Platinum Dataset

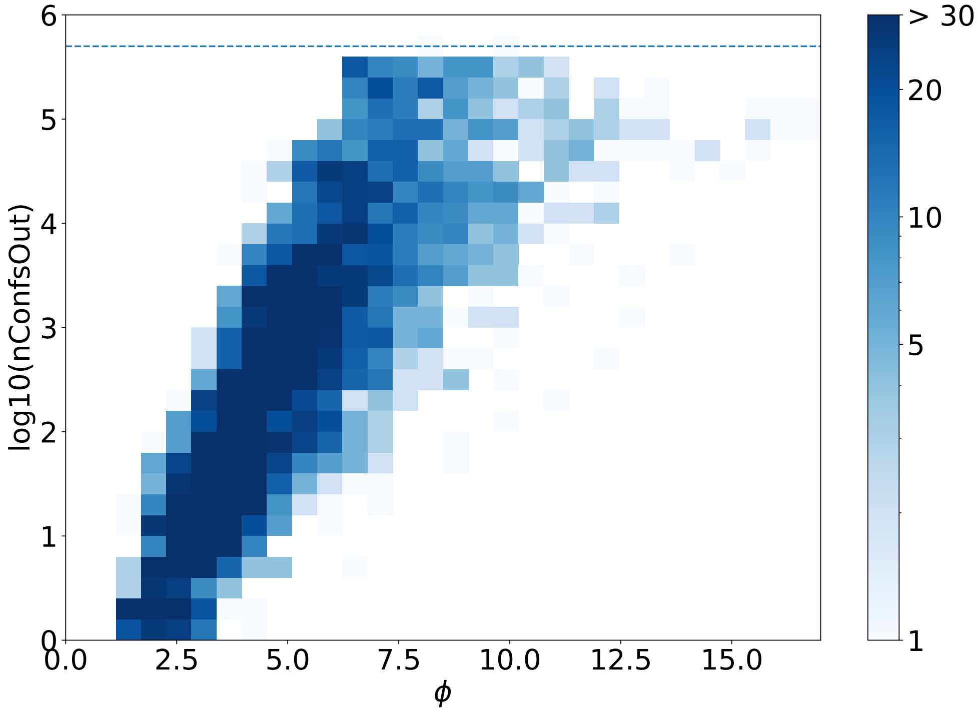

Figure S8: Correlation between the Kier ϕ index and the logarithm of the number of conformers in the pruned ensembles

S7 Classification Comparison between Heavy-Atom RMSD and TFD

Figure S9 shows the results when conducting the same analysis as introduced above, but instead of comparing the ground truth (RMSD at chosen threshold) against a TABS categorization, comparing against

the categorization in same and different by TFD with a threshold of 0.2 as the value is the proposed threshold by Schulz-Gasch [10]. The NPV and PPV shown in Figure S9 demonstrate that classifications in same and different for two conformers over all molecule ensembles from the Platinum set are not the same for the two measures of RMSD and TFD.

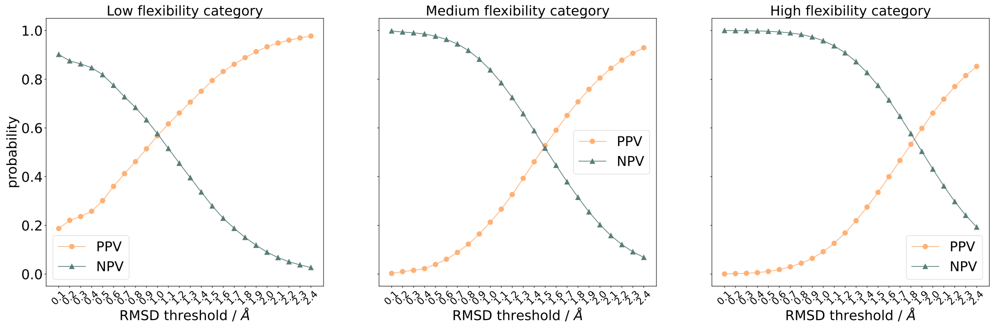

Figure S9: Ensemble categorization according to RMSD vs TFD showing positive predictive values (PPV) and negative predicitive values (NPV) for varying RMSD thresholds sorted into the different flexibility categories according to the introduced nTABS categorization, the TFD categorization is kept constant with a threshold of 0.2

While the classifications based on TABS and RMSD were reaching NPV and PPV values of over 80 % for all three flexibility categories, the RMSD vs TFD comparison do not exceeded 60 %.

S8 Performance Measurements

n	t / ms
100	177
1000	1330
10000	15100

Table S1: Cyclohexane ensemble sizes n and the timings to calculate the TABS labels.

References

[1] RDKit github repo, rotatable bond. `https://github.com/rdkit/rdkit/blob/master/Code/GraphMol/Descriptors/Lipinski.cpp`, Acessed: 2024-03-04.

[2] Allen, F. H. The Cambridge Structural Database: a quarter of a million crystal structures and rising. *Acta Crystallogr., Sect. B: Struct. Sci.* **2002**, *58*, 380–388.

[3] Groom, C. R.; Allen, F. H. The Cambridge Structural Database in Retrospect and Prospect. *Angew. Chem. Int. Ed.* **2014**, *53*, 662–671.

[4] Schärfer, C.; Schulz-Gasch, T.; Ehrlich, H.-C.; Guba, W.; Rarey, M.; Stahl, M. Torsion Angle Preferences in Druglike Chemical Space: A Comprehensive Guide. *J. Med. Chem.* **2013**, *56*, 2016–2028.

[5] Guba, W.; Meyder, A.; Rarey, M.; Hert, J. Torsion Library Reloaded: A New Version of Expert-Derived SMARTS Rules for Assessing Conformations of Small Molecules. *J. Chem. Inf. Model.* **2016**, *56*, 1–5.

[6] Riniker, S.; Landrum, G. A. Better Informed Distance Geometry: Using What We Know To Improve Conformation Generation. *J. Chem. Inf. Model.* **2015**, *55*, 2562–2574.

[7] Wang, S.; Witek, J.; Landrum, G. A.; Riniker, S. Improving Conformer Generation for Small Rings and Macrocycles Based on Distance Geometry and Experimental Torsional-Angle Preferences. *J. Chem. Inf. Model.* **2020**, *60*, 2044–2058.

[8] RDKit github repo, torsion preferences. `https://github.com/rdkit/rdkit/blob/master/Code/GraphMol/ForceFieldHelpers/CrystalFF/torsionPreferences_v2.in`, Accessed: 2023-08-21.

[9] Dixon, D. A.; Komornicki, A. Ab initio conformational analysis of cyclohexane. *J. Phys. Chem.* **1990**, *94*, 5630–5636.

[10] Schulz-Gasch, T.; Schärfer, C.; Guba, W.; Rarey, M. TFD: Torsion Fingerprints As a New Measure To Compare Small Molecule Conformations. *J. Chem. Inf. Model.* **2012**, *52*, 1499–1512.
